# Thermally Driven Membrane Phase Transitions Enable Content Reshuffling in Primitive Cells

**DOI:** 10.1021/jacs.1c06595

**Published:** 2021-10-01

**Authors:** Roger Rubio-Sánchez, Derek K. O’Flaherty, Anna Wang, Francesca Coscia, Gianluca Petris, Lorenzo Di Michele, Pietro Cicuta, Claudia Bonfio

**Affiliations:** † Biological and Soft Systems, Cavendish Laboratory, University of Cambridge, Cambridge CB3 0HE, U.K.; ‡ Department of Chemistry, University of Guelph, Guelph ON N1G 1Y4, Canada; § School of Chemistry, University of New South Wales, Sydney, New South Wales 2052, Australia; ∥ Medical Research Council Laboratory of Molecular Biology, Cambridge Biomedical Campus, Cambridge CB2 0QH, U.K.; ⊥ Fondazione Human Technopole, Structural Biology Research Centre, Milan 20157, Italy; # Department of Chemistry and fabriCELL, Imperial College London, Molecular Sciences Research Hub, London W12 0BZ, U.K.; ¶ Yusuf Hamied Department of Chemistry, University of Cambridge, Cambridge CB2 1EW, U.K.

## Abstract

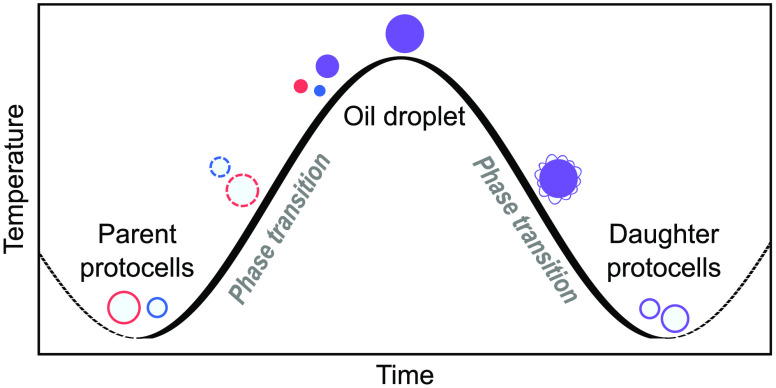

Self-assembling single-chain amphiphiles available in the prebiotic environment likely played a fundamental role in the advent of primitive cell cycles. However, the instability of prebiotic fatty acid-based membranes to temperature and pH seems to suggest that primitive cells could only host prebiotically relevant processes in a narrow range of nonfluctuating environmental conditions. Here we propose that membrane phase transitions, driven by environmental fluctuations, enabled the generation of daughter protocells with reshuffled content. A reversible membrane-to-oil phase transition accounts for the dissolution of fatty acid-based vesicles at high temperatures and the concomitant release of protocellular content. At low temperatures, fatty acid bilayers reassemble and encapsulate reshuffled material in a new cohort of protocells. Notably, we find that our disassembly/reassembly cycle drives the emergence of functional RNA-containing primitive cells from parent nonfunctional compartments. Thus, by exploiting the intrinsic instability of prebiotic fatty acid vesicles, our results point at an environmentally driven tunable prebiotic process, which supports the release and reshuffling of oligonucleotides and membrane components, potentially leading to a new generation of protocells with superior traits. In the absence of protocellular transport machinery, the environmentally driven disassembly/assembly cycle proposed herein would have plausibly supported protocellular content reshuffling transmitted to primitive cell progeny, hinting at a potential mechanism important to initiate Darwinian evolution of early life forms.

## Introduction

The emergence of primitive cell cycles represents a step toward the generation of model protocells. Inspired by modern biology, biochemists and synthetic biologists aiming to mimic evolution have long tried to understand how minimal cells could recursively undergo *model* cell cycles, often driven by steps of growth and division,^1−3^ while continuing to sustain compartmentalized biochemical processes. One unresolved difficulty with proposing *primitive* cell cycles is that prebiotic components should be employed, and the identified model conditions should be compatible with the prebiotic environment and processes.

Among the prebiotically plausible biological building blocks, fatty acids were likely major components of primitive membranes.^4−7^ Recent reports have demonstrated how fatty acid-based compartments can host prebiotically relevant reactions, including activation of building blocks,^8^ metal-based catalysis,^9^ and nonenzymatic RNA replication.^5,6,10^ However, the limited stability of fatty acid-based protocells to changes in temperature, pH, and ionic strength^11−13^ needs to be taken into account when one aims at expanding the repertoire of compartmentalized prebiotic processes and identifying plausible pathways of primitive cell replication.

Fatty acid-based protocells have been shown to undergo cycles of growth and division.^14−16^ Specifically, the growth of large multilamellar vesicles can be achieved by addition of fatty acid micelles, resulting in long thread-like bilayer structures that, upon shearing, divide into smaller vesicles with no significant loss of protocellular content.^16^ Such a model of primitive cell cycle relies on two assumptions: (i) that the required environmental conditions are not subject to fluctuations, as not to destabilize fatty acid vesicles, and (ii) that content reshuffling, product dilution, or substrate uptake is not required for encapsulated prebiotic reactions to work. However, natural temperature and pH gradients would have easily formed both in sunlit aqueous surfaces and in hydrothermal environments on early Earth^17−19^ and, likely, would have been required to support most prebiotic (bio)chemical processes, including the synthesis^20^ and polymerization^21,22^ of life’s building blocks. Also, in the absence of modern trafficking biosystems to rely upon, accumulation of products or lack of substrates could have inhibited compartmentalized protocellular processes.^23,24^

In this study, we unravel a new, prebiotically plausible mechanism, in which thermal variations drive the formation of new cohorts of fatty acid-based protocells and the reshuffling of their encapsulated material. At high temperatures, unilamellar vesicles collapse at first into multilayered structures, then into lipid droplets, concomitantly releasing their protocellular content. At low temperatures, the self-assembly of fatty acid protocells via membrane budding from the surface of lipid droplets yields daughter protocells with re-encapsulated protocellular material. Such lipid phase transitions, triggered by thermally induced pH fluctuations, can be modulated by the presence of relevant building blocks, like nucleotides and peptides. Thus, by exploiting the thermal instability of prebiotically relevant lipid vesicles, our work proposes a novel geochemically plausible primitive mechanism, underpinned by the reversible pH-driven assembly and disassembly of the protocellular state, and addresses for the first time the fundamental role of protocellular content mixing and reshuffling. Importantly, the versatile behavior of fatty acids in response to temperature and pH observed herein greatly promotes their employment in colloid chemistry and soft matter research for the design of environmentally responsive self-assembled (bio)materials. Here we show to which extent the choice of the hydrating buffer and the effect of chemical additives (i.e., nucleotides, peptides, and salts) play a fundamental role in the occurrence, reversibility, and tunability of fatty acid phase transitions, thus better understanding and expanding the physicochemical space in which fatty acid aggregates can be studied and employed.

## Results

### Reversible pH-Driven Lipid Phase Transitions

Moderate thermal gradients, such as those naturally generated in hydrothermal environments or sunlit shallow ponds, were shown to support the amplification of functional nucleic acid strands,^25,26^ as well as the accumulation and self-assembly of prebiotic amphiphiles (between 5 and 50 °C).^27^ However, previous studies on the permeability of primitive cells showed that loaded vesicles, made of short- to medium-chain fatty acids, do not retain oligonucleotides when exposed to high temperatures (above 50 °C).^13^ While heat-stable lipid mixtures were identified, the reported thermal instability of certain prebiotic vesicles begs the following question: What mechanism could cause the release of the encapsulated content at high temperatures?

**Figure 1:**
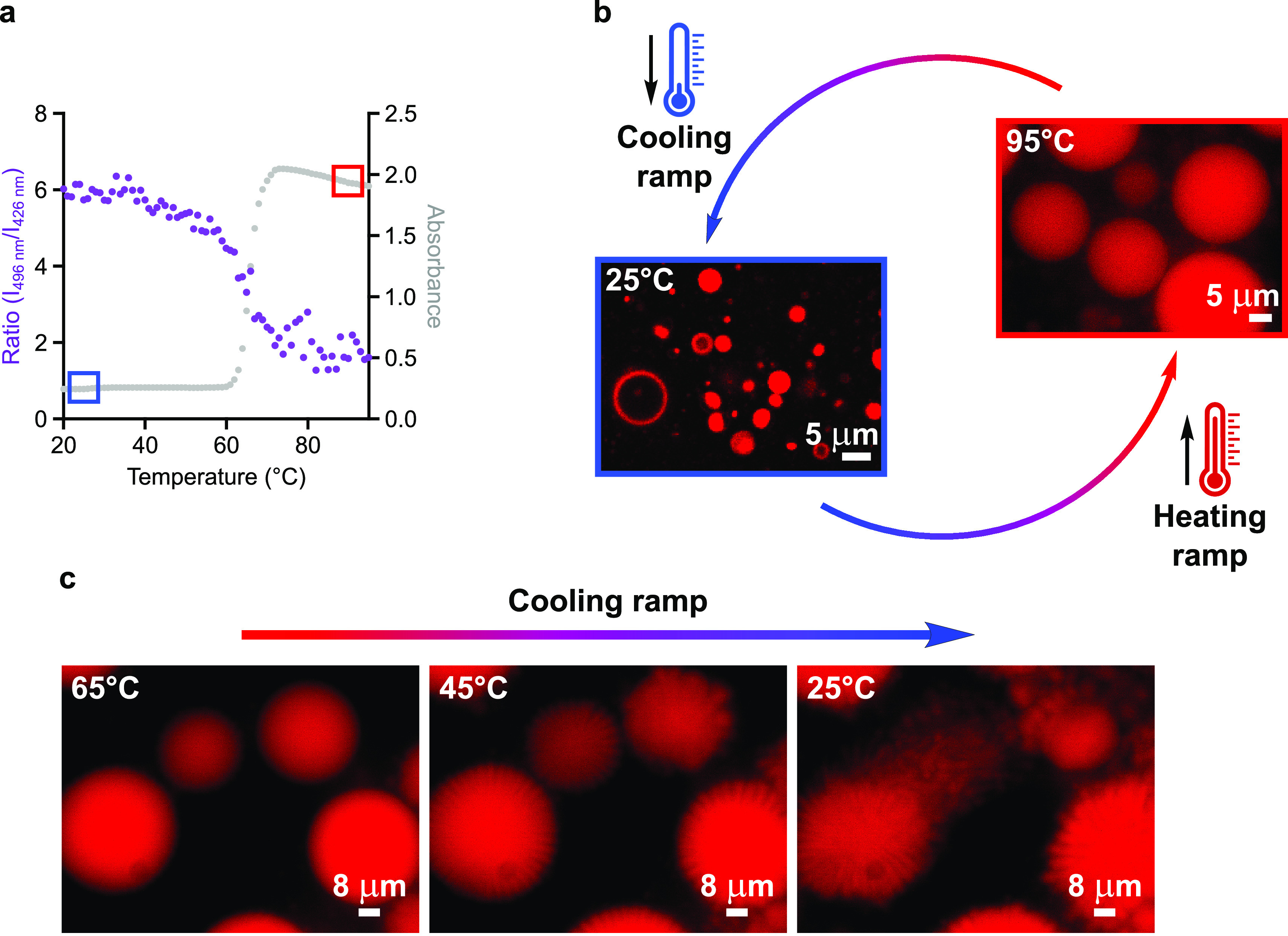
Thermal cycling drives reversible and tunable fatty acid phase transitions. (a) Overlapped profiles of turbidity and fluorescence intensity as a function of temperature for 100-nm-radius vesicles made of 50 mM myristoleic acid (in 200 mM Tris-HCl, pH 7, 25 °C → 95 °C). For fluorescence experiments, Laurdan (20 μM) was embedded in the lipid bilayer. Absorbance (gray) is monitored at 420 nm, whereas Laurdan fluorescence (purple) is monitored at λ_exc_ = 350 nm, λ_em_ = 426 and 496 nm. (b) Schematic representation of thermal cycles explored in this study. Microscopy images after the heating ramp (95 °C, hot-stage epifluorescence microscopy images) and after the cooling ramp (25 °C, confocal microscopy images) are shown for myristoleic acid vesicles. (c) Membrane budding observed during the cooling ramp on the surface of myristoleic acid droplets by hot-stage epifluorescence microscopy. *n* = 3 for data in (a).

To shed light on the behavior of fatty acid vesicles upon heat exposure, we sought to explore whether alterations in membrane morphology might occur and be responsible for the leakage of the encapsulated content at high temperatures. A buffered solution containing extruded myristoleic acid vesicles was heated to 95 °C, and changes in lipid packing and turbidity were detected by fluorescence and UV–vis spectroscopy. Myristoleic acid was chosen as a proxy for self-assembling amphiphiles.^10^ For fluorescence experiments, the lipophilic probe Laurdan was embedded in the lipid bilayer to provide information on changes in lipid packing and membrane dynamics.^14^ Notably, at high temperatures we observed a sharp increase in turbidity (420 nm, both in the presence and in the absence of Laurdan), concurrent with a gradual decrease in the fluorescence intensity ratio of Laurdan emission maxima (496/426 nm) (Figures 1a and S1a). Remarkably, vesicles prepared with prebiotically relevant amphiphile mixtures^4^ (decanoic acid and decanol, 2:1 ratio) showed similar absorbance and fluorescence profiles (Figure S1b,c). Control experiments in isothermal conditions and in a range of pH values were performed with Laurdan-labeled vesicles to fully support our findings (Figure S2). That is, our fluorescence and absorbance data point toward a major heat-driven alteration in lipid packing and membrane morphology.

To identify the cause of increased turbidity, we modeled^28^ the expected light-scattering profile for extruded unilamellar vesicles upon heat exposure. The computed optical density value is in perfect agreement with our experimental observations for decanoic acid-based vesicles (Figure S3). Even though a small increase in optical density could be accounted for by an increase in either vesicle radius or lamellarity (Figure S3a,b), the observed increase of absorbance above 0.5 can only be attributed to the formation of larger dense structures with high internal lipid fraction (Figure S3c). Our model thus suggests that the observed heat-induced increase in the size of lipid particles is accompanied by a variation in lipid density. In order to elucidate the effect of heating on the morphology of fatty acid membranes, we turned to hot-stage epifluorescence microscopy. Along the heating ramp (25 °C → 95 °C, at a rate of 0.1 °C·s^–1^), myristoleic acid vesicles exhibit an increasingly dynamic behavior (Movie S1) and eventually collapse into micron-sized lipid droplets at high temperatures (Figures 1b and S4). Kept at 95 °C for 15 min, fatty acid droplets coalesce into larger structures. Then, upon cooling to 25 °C, membrane budding from the water–oil interface results in the generation of daughter fatty acid vesicles (Figure 1c). These findings are consistent with both our modeling and experimental spectroscopic data.

As illustrated in their phase diagrams, fatty acids form membranes spontaneously in aqueous solutions at room temperature, in a narrow pH window around their apparent p*K*_a_.^29^ In isothermal conditions and high pH, fatty acids are fully deprotonated and form micelles, whereas, at low pH, they are fully protonated and form an oil phase.^29^ Additional lamellar structures, as well as cubic and hexagonal phases, are observed at intermediate pH values or higher lipid content.^30,31^ As the p*K*_a_ of most functional groups varies appreciably with temperature,^32^ the reversible phase transition observed for myristoleic acid vesicles likely results from pH fluctuations during heating and cooling ramps. In further support of our hypothesis, the temperature-dependence of pH for a number of biological buffers was evaluated by means of UV–vis spectroscopy in the presence of fluorescein, a pH-sensitive probe (Figure S5). While the p*K*_a_ of phosphate was only mildly affected by heat, the pH of solutions containing Tris-HCl dropped by two units when heated from 25 °C to 95 °C. Such results suggest that myristoleic acid vesicles prepared in Tris-HCl buffer are destabilized upon heating, i.e., when the pH becomes too acidic (below 6.5), and convert to lipid droplets. At high temperatures, the local increase in interfacial tension, due to the high concentration of protonated fatty acids, likely leads to the high-energy lipid surfaces coalescing into larger droplets. Upon cooling, as deprotonation of fatty acids in the outermost leaflet of lipid droplets could lead to increasing curvature, undulating folds occur on the droplet surface, resulting in membrane budding.

### Tunable Disassembly and Reassembly of Lipid Vesicles

A series of experiments was performed to interrogate lipid dynamics and phase transitions systematically for both myristoleic acid- and decanoic acid-based membranes under different conditions. The reversible fatty acid vesicle-to-lipid droplet conversion occurs in most biological buffers, and in combinations thereof, as well as in self-buffering conditions, thus indicating that such lipid phase transitions could have naturally occurred in a wide range of primordial buffered and unbuffered environments (Figures S6–S8). To explore the tunability of phase-transition temperatures, variations of initial pH and ionic strength values, buffer concentrations, and fatty acid composition and concentrations were also tested (Figure S9–S13). Intriguingly, the addition of peptides and nucleotides, as prebiotically relevant building blocks, shifts the transition temperature to lower values in a charge-dependent manner, likely due to ionic interactions with lipid headgroups affecting the bilayer’s hydrogen bonding network (Figure S10). These prebiotic additives did not seem to affect further the size and lamellarity of primitive membranes upon thermal cycling, in contrast with recent observations on fatty acid-based vesicles undergoing wet–dry cycles in the presence of single amino acids.^33^ Such divergent, yet intriguing findings on the effect of amino acids and peptides on the thermal stability of fatty acid-based vesicles should be further explored in future work. Overall, these results indicate that temperature gradients generate pH fluctuations in most buffered environments and in a wide range of lipid and buffer concentrations, as well as in the absence of buffers, and drive reversible fatty acid phase transitions, which can be modulated by the presence of life’s biological building blocks.

**Figure 2:**
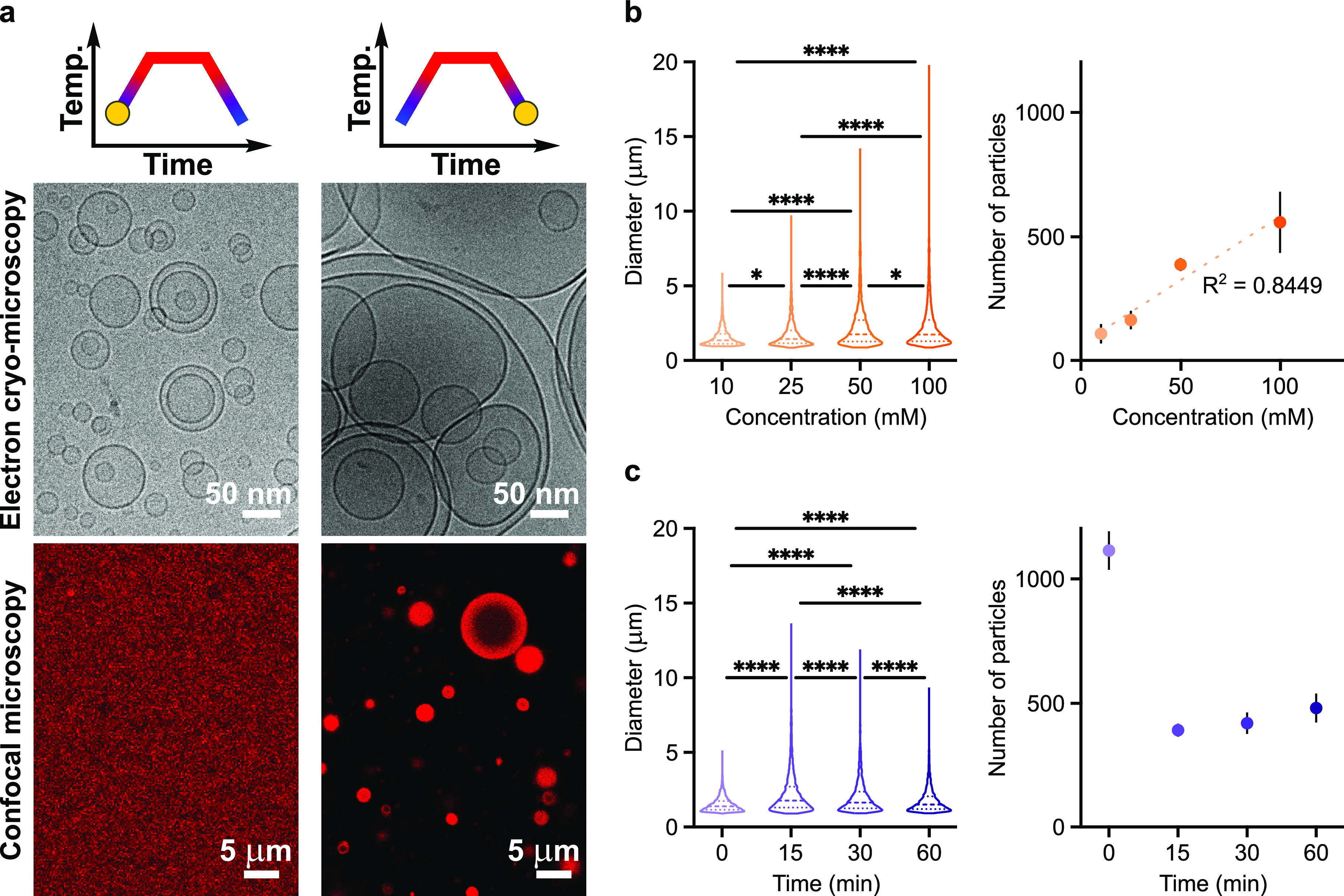
Thermal cycling drives disassembly and reassembly of fatty acid membranes. (a) Electron cryo-microscopy (top) and confocal microscopy (bottom) images collected before and after thermal cycles for myristoleic acid vesicles. (b) Analyses of myristoleic acid samples showing how the size (left) and number (right) of the next-generation population of vesicles are affected by lipid concentration. (c) Analyses of independently prepared myristoleic acid samples showing how the size (left) and number (right) of regenerated vesicles are affected by heating time. Analyses in (b) and (c) were performed on collected confocal microscopy images for 100-nm-radius vesicles made of 50 mM myristoleic acid vesicles in 200 mM Tris-HCl, pH 8, after thermal cycling. Upon cooling the samples to 25 °C, vesicles were allowed to re-equilibrate for 1 h. Minimal diameter cutoff for image processing = 500 nm. Statistical significance was assessed using the one-way ANOVA test, *n* = 10 independent experiments. Statistical values obtained for *P = < 0.05, ****P = < 0.0001. Center dashed line represents median; dotted lines represent upper and lower quartiles. *n* = 3 for data in (a).

The spontaneous self-assembly of fatty acid membranes generally results in polydisperse multilamellar structures and, from those, unilamellar vesicles are then formed by extrusion.^29^Our confocal and electron cryo-microscopy data reveal that, when 50 mM extruded small unilamellar myristoleic acid vesicles undergo thermal cycling (25 °C → 95 °C → 25 °C, at a rate of 0.1 °C·s^–1^), large multilamellar structures are formed via membrane budding from lipid droplets (Figure 2a). However, the extrusion of fatty acid vesicles is not required to observe reversible temperature-induced phase transitions. Notably, when a lower lipid concentration was employed (10 mM), we observed smaller fatty acid droplets and multilamellar vesicles, after heating and cooling ramps, respectively (Movie S2 and Figure S14). Hence, we performed a statistical analysis on the newly generated multilamellar structures as a function of initial lipid concentration and vesicle size and incubation time at high temperature (Figures 2b,c and S15). Our data, obtained on 10 independent experiments, show that higher lipid concentrations and longer incubation times at high temperature result in larger fatty acid droplets; when fatty acid vesicles are only briefly exposed to heat, small lipid droplets do not have enough time to coalesce and thus generate small multilamellar structures upon cooling. At low temperatures, more numerous vesicles, larger in size, can be generated only if lipid droplets are provided sufficient time for efficient membrane budding and self-assembly (Figure S16). Interestingly, the initial diameter of vesicles does not play a significant role in fatty acid droplet formation or size, nor does it affect the reversibility of vesicle-oil droplet conversion, and thus has no effect on the size or number of daughter myristoleic acid vesicles (Figure S17a,b). Moreover, exposing a solution of myristoleic acid vesicles to multiple thermal cycles did not affect the number or size of newly formed multilamellar vesicles (Figure S17c). Such findings further demonstrate that the initial lipid concentration, the size of lipid droplets, and the incubation time are the main parameters responsible for the number and average size of daughter protocells. Similar results were obtained for decanoic acid-based vesicles (Figure S18). Overall, our findings suggest that lipid phase transitions, which are controlled by temperature-driven pH changes, could be exploited to circumvent bilayer fusion barriers. Upon heat exposure, the lipid system transitions from a colloidally stable bilayer dispersion, which is dominated by fusion barriers, to a dispersion of oil droplets, which is dominated by interfacial tension (and hence favors coalescence). Importantly, the reversible and tunable pH-induced fatty acid phase transitions described herein support the disassembly and reassembly of lipid vesicles of varied size and quantity. A critical question to be answered is whether such a simple and robust phenomenon, likely to have occurred recursively and under relatively mild conditions on early Earth, may have supported the onset of a primitive cell cycle.

### Release and Re-encapsulation of the Protocellular Content

**Figure 3:**
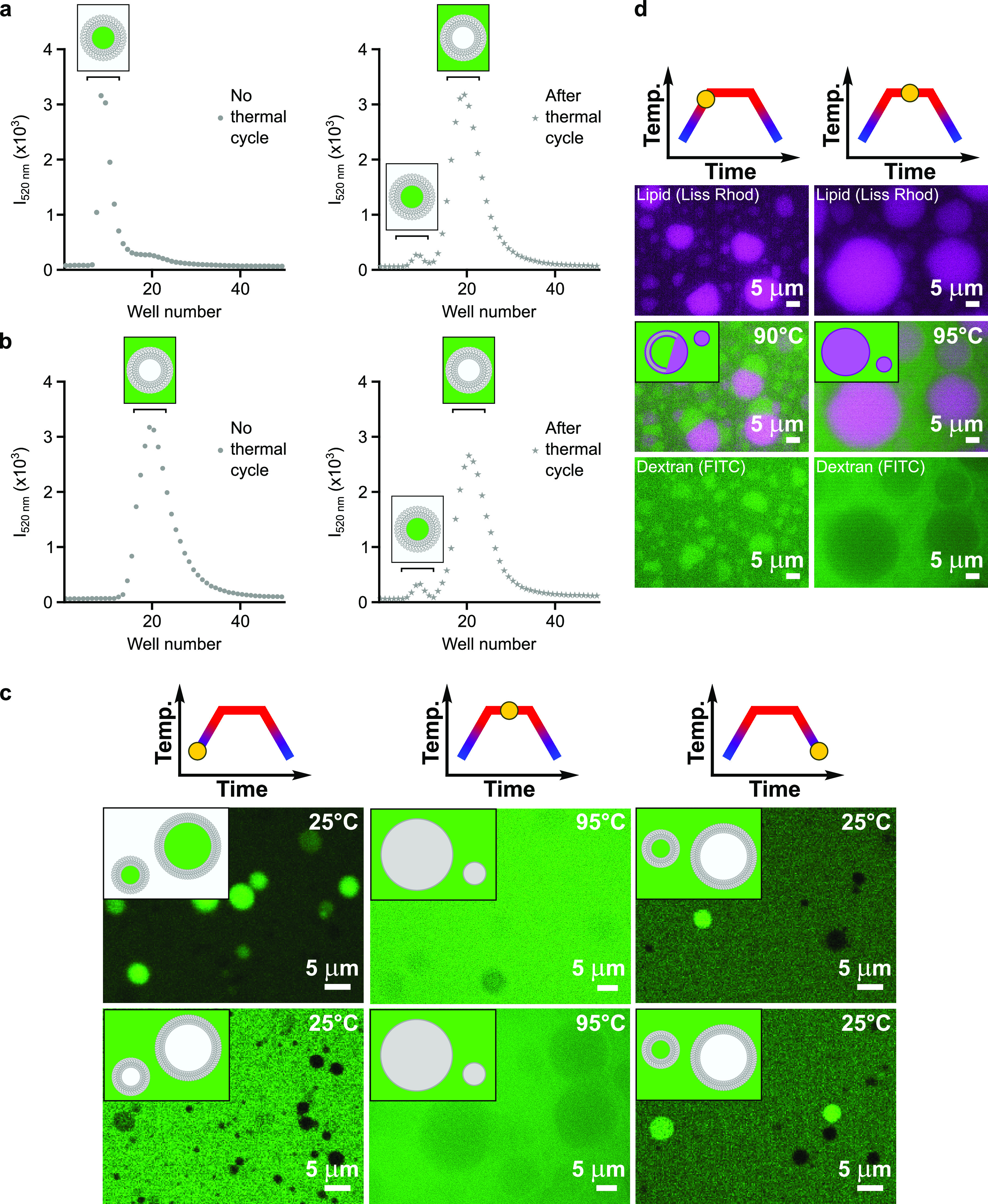
Thermal cycling drives release and re-encapsulation of protocellular content. (a) Size-exclusion chromatograms showing partial re-encapsulation (3.2%) of FITC-dextran in 100-nm-radius vesicles made of 50 mM myristoleic acid in 200 mM Tris-HCl, pH 8, upon heat exposure. Content uptake was monitored by fluorescence (λ_exc_ = 495 nm). (b) Size-exclusion chromatograms showing partial encapsulation (4.1%) of FITC-dextran in 100-nm-radius vesicles made of 50 mM myristoleic acid in 200 mM Tris-HCl, pH 8, upon heat exposure. Content uptake was monitored by fluorescence (λ_exc_ = 495 nm). (c) Microscopy images corresponding to experiments reported in (a) (top row) and (b) (bottom row) before the thermal cycles (25 °C, confocal microscopy images), after the heating ramps (95 °C, hot-stage epifluorescence microscopy images) and after the cooling ramps (25 °C, confocal microscopy images) are shown for 50 mM myristoleic acid vesicles in 200 mM Tris-HCl, pH 8. (d) Hot-stage epifluorescence microscopy images corresponding to the experiment reported in (a) at high temperature values (90 °C, left; 95 °C, right) show the formation of faceted myristoleic acid structures (90 °C, left), which transiently trap the aqueous content. Such multilayer structures then convert to lipid droplets (95 °C, right), completely releasing the encapsulated material. Samples for confocal microscopy analyses were diluted after thermal cycling to reduce the background noise of the unencapsulated fluorescent material. Data are mean and SEM, *n* = 3 independent experiments.

The thermally induced vesicle-to-lipid droplet phase transition observed herein explains why fatty acid protocells do not retain compartmentalized oligonucleotides when exposed to high temperatures,^13^ as demonstrated by the specular features of turbidity and leakage profiles (Figure S19). We thus investigated whether the next-generation population of vesicles, formed upon cooling via membrane budding from lipid droplets, could re-encapsulate the released content. Myristoleic acid vesicles containing fluorescently labeled dextran (5 kDa), chosen as a model cargo, were either kept at room temperature or subject to a full thermal cycle (25 °C → 95 °C → 25 °C, at a rate of 0.1 °C·s^–1^), then loaded onto a size-exclusion column to evaluate the loss of compartmentalized material (Figure 3a). While most of the fluorescent content (96.8%) was released from vesicles upon heat exposure as previously reported,^13^ a peak corresponding to compartmentalized dextran (3.2%) could be detected at the end of the thermal cycle. There are two possible explanations for such a finding: (i) a small fraction of the fatty acid vesicles is not destroyed by the thermal cycle or (ii) the fluorescent content is entirely released during the heating ramp and partially re-encapsulated during the reassembly of membranes upon cooling. Scenario (ii) was confirmed by running the experiment with initially empty vesicles and unencapsulated fluorescently labeled dextran (Figure 3b). Here, dextran-loaded vesicles were observed only after the thermal cycle, demonstrating that encapsulation takes place during vesicle regeneration. While the possibility of a few vesicles to survive to thermal cycles cannot be completely ruled out, our data suggest that the release and re-encapsulation of protocellular content is majorly related to the reversible pH-driven phase transition between fatty acid vesicles and oil droplets.

Permeability studies performed on phospholipid-based vesicles^34^ demonstrate how transient pore formation is responsible for increased membrane permeability around and above the chain melting transition temperature. However, the vesicle-to-oil droplet transition, observed for fatty acid vesicles in temperature-dependent buffers, seems to occur concomitantly with the release of aqueous content. To gain biophysical insight on the lipid droplet-to-vesicle phase transition and the related content re-encapsulation, we repeated both experiments, visualizing the process by confocal and hot-stage epifluorescence microscopy (Figure 3c). The mechanism we are proposing is that, when the solution becomes too acidic, unilamellar vesicles begin to collapse at first into multilamellar structures still capable of hosting aqueous milieus and later into highly dense lipid droplets, which expel the protocellular content to the bulk.^29−31^ The observation of faceted structures at high temperatures suggests that the encapsulated fluorescent content is not immediately released in solution, but rather transiently trapped by (multiple) lipid bilayers (Figure 3d). Upon further heat exposure or temperature increase, the encapsulated material is completely excluded, and lipid droplets appear as dark circular spots in the fluorescent background. Along the cooling ramp, as the pH value becomes more suitable for stabilizing fatty acid vesicles, the lipid molecules that are present at the surface of the fatty acid droplets begin to self-organize into multilamellar structures (Figure S20) with intercalated fluorescent aqueous content. Thermally induced pH fluctuations are thus responsible not only for the disassembly and reassembly of primitive compartments but also for both the release and subsequent reuptake of protocellular material.

### Content Mixing and Reshuffling: A New Cohort of Protocells

**Figure 4:**
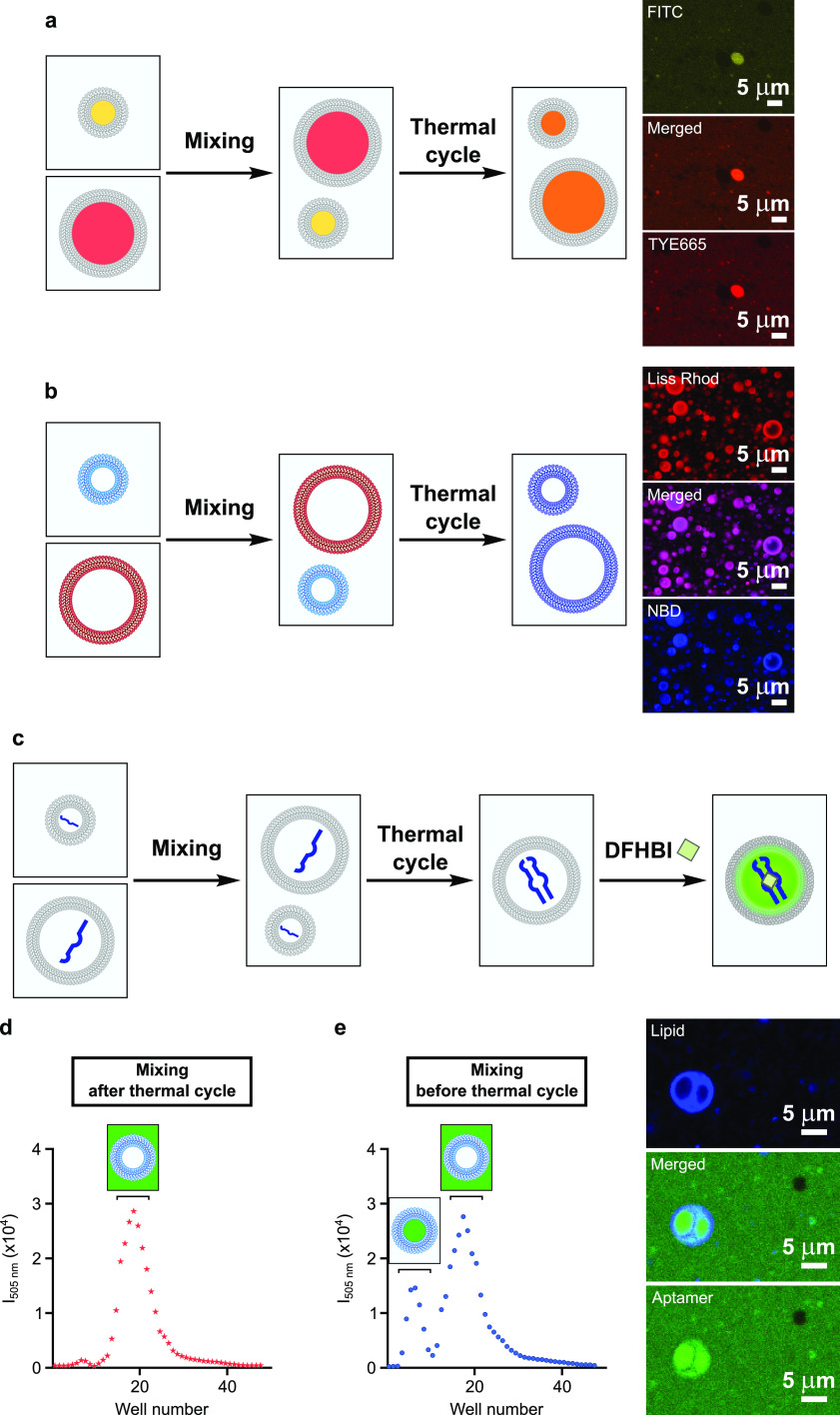
Thermal cycling drives reassembly of protocells with reshuffled membrane material and encapsulated content. (a) Schematic representation and confocal microscopy images for experiments with populations of vesicles with different fluorescent content. 100-nm-radius vesicles, made of 50 mM myristoleic acid in 200 mM Tris-HCl, pH 8, and containing either FITC-10nt or TYE665-10nt oligonucleotides, were mixed before undergoing thermal cycling and, after 1 h re-equilibration at room temperature, were visualized by confocal microscopy. (b) Schematic representation and confocal microscopy images for experiments with populations of vesicles, labeled with different fluorescent lipids. 100-nm-radius vesicles, made of 50 mM myristoleic acid in 200 mM Tris-HCl, pH 8, and either NBD-PE or Rh-DHPE, were mixed before undergoing thermal cycling and, after 1 h re-equilibration at room temperature, were visualized by confocal microscopy. (c) Schematic representation of the experiment to reconstitute a split Broccoli aptamer inside myristoleic acid vesicles. (d) Size-exclusion chromatograms showing no reconstitution of the split Broccoli aptamer when vesicle mixing occurs after thermal cycling. (e) Size-exclusion chromatograms showing reconstitution of the split Broccoli aptamer (20.3%) when vesicle mixing occurs before thermal cycling. Experiments for Broccoli aptamer reconstitution were performed with 100-nm-radius vesicles made of 50 mM myristoleic acid in 200 mM Tris-HCl, pH 8. Broccoli aptamer reconstitution was monitored by fluorescence of DFHBI (λ_exc_ = 505 nm). Confocal images were collected prior to purification. Data are mean and SEM, *n* = 3 independent experiments.

One could imagine that early Earth was inhabited by different populations of protocells, each hosting their own prebiotic content, including genetic material. Those protocells would have likely been exposed to naturally occurring pH and temperature gradients^17^ and, when made of short- to medium-chain fatty acids, would have undergone the phase transitions discussed in the previous sections. Crucially, when two distinct fatty acid protocell populations undergo such a phase transition, both their encapsulated material and lipids can get mixed by virtue of the assembly/disassembly cycles of their building blocks. Therefore, we propose thermal cycling as a plausible means to potentially generate a new population of protocells with reshuffled content and bilayer components. To investigate this hypothesis, we prepared empty fatty acid protocells and exposed them to three sequential thermal cycles, adding a different fluorescent oligonucleotide to the solution before every cycle. FITC-, Cy3-, and Cy5.5-labeled 10-nucleotide oligomers were selected as proxy for protocellular genetic content. Fluorescence profiles recorded after size-exclusion column chromatography suggest that fluorescent oligonucleotides are re-encapsulated in the second-, third-, and fourth-generation of daughter protocells, respectively (Figure S21). Such a result further demonstrates the reversibility of membrane assembly and disassembly, as well as content release and uptake, upon recurring thermal cycling. Importantly, it has been previously shown that RNA degradation does not occur when short RNAs are exposed to high temperatures at neutral pH for short amounts of time;^22^ thus, we do not expect nucleic acid degradation upon thermal cycling. Next, we prepared two distinct vesicle populations, each loaded with a different fluorophore, and mixed them prior to incubation at high temperature; newly generated vesicles exhibited both fluorescence signals, confirming that the encapsulation of reshuffled content took place (Figure S22). Confocal microscopy images confirm that both fluorescent oligonucleotides are co-compartmentalized within the same protocells (Figure 4a). As thermally driven phase transitions yield new populations of daughter fatty acid protocells, we sought to explore whether new blended membranes would also be generated by lipid mixing in the oil phase. Large mixed multilamellar structures were observed upon heating from combinations of fluorescently labeled unilamellar protocells (Figure 4b). No content nor lipid reshuffling could be detected for populations of vesicles only mixed after thermal cycling (Figures S23 and S24).

A likely interesting advantage of protocellular reshuffling is the potential for a new cohort of primitive compartments to arise with novel functionalities. While most of the previously encapsulated material is not taken in by the daughter cells, a low re-encapsulation efficiency might have still been sufficient for some primitive cells to achieve life-like functionalities. Additionally, certain processes may have required a dilution step such that feedback inhibition could be suppressed. For example, mixing oligonucleotides that are independently synthesized within distinct fatty acid vesicles could give rise to new rounds of nucleic acid polymerization, as well as ribozyme-based catalytic processes. As a proof-of-concept, we designed a set of experiments aimed at reconstituting a shorter split version of the Broccoli aptamer^35^ within the new population of protocells, generated via thermal cycling (Figure 4c). When two populations of purified vesicles, each loaded with a different fragment of the selected aptamer, are first exposed to thermal ramps and later mixed, no fluorescence from the encapsulated DFHBI fluorogen can be detected (Figure 4d). However, when a mixed sample of different protocells undergoes thermal cycling, the newly generated vesicles incorporate both RNA strands, thus efficiently assembling the Broccoli aptamer, which enhances the fluorescent signal of the DFHBI fluorogen (Figure 4e). Together, these results provide strong evidence that thermally driven pH variations can drive reversible and tunable lipid phase transitions and support the release and reshuffling of protocellular content, thus providing a prebiotically plausible possible mechanism for the emergence of a new generation of protocells with potentially enhanced functionalities.

## Discussion

In the absence of modern biological machinery, primitive cells likely had to rely on the self-assembling and dynamic properties of their components and on interactions with their environment to achieve basic cellular functions, including primitive cellular replication. Prebiotically plausible cycles involving alternating dehydration and rehydration steps have been recently proposed;^36^ however, volatile reactants can easily escape and pH- or temperature-sensitive substrates can undergo potentially irreversible degradation when prolongedly exposed to high temperatures. Alternatively, primitive cell cycles could be based on recursive growth and division of lipid vesicles.^11^ Despite better mimicking modern cellular pathways, such iterations would not support the uptake, release, and exchange of protocellular content with the environment.

Single-tailed lipids, such as those employed herein, are known to exist in a broader range of phases^29^ compared to modern-day phospholipids. However, in the studies on prebiotic single-tailed amphiphiles and membranes derived thereof, these other phases have rarely been accessed or explored for their potential role in prebiotic physicochemical pathways. Our work contributes to filling this knowledge void by exploring such a prebiotic lipid-phase space, and taking advantage of it. Importantly, although medium-chain unsaturated lipids, such as myristoleic acid, have not been yet synthesized in prebiotic conditions, we demonstrate that reversible phase transitions can be similarly achieved and modulated with prebiotically relevant lipid mixtures,^4^ such as those containing decanoic acid.

Reports on the thermal instability of fatty acid protocells^13^ led to the hypothesis that the observed leakage of encapsulated genetic material was due to exposure to high temperatures, causing a concomitant increase in membrane permeability. In parallel, the observation that small unilamellar vesicles would convert into large multilamellar vesicles upon heating–cooling cycles was attributed to heat-induced fusion events.^37^ Our work now clarifies these misunderstood phenomena, demonstrating that the triggering of a lipid phase transition, rather than improved permeability, is the cause of content release. Moreover, our results show that pH fluctuations occurring in temperature-sensitive buffers, rather than temperature variations themselves, induce lipid phase transitions and hence leakage of encapsulated material. Interestingly, we have shown, like others,^38^ that temperature-dependence buffering capacity varies from one buffer to another. As such, prebiotic mechanisms described herein would unquestionably depend on the identity and availability of prebiotic buffers. Finally, we have shown that membrane reassembly after condensing into an oil phase, rather than fusion of lipid vesicles, spontaneously yields large multilamellar vesicles upon a heating–cooling cycle. The formation of an intermediate lipid droplet phase is what enables and explains all these observations. Our thorough and refined understanding and mechanistic explanation of membrane dynamics and the cargo release mechanism have led to the central discovery herein described and characterized, the reshuffling/re-encapsulation pathway.

Thermally and pH-responsive lipid supramolecular systems, such as those observed and characterized herein, have also a wide range of important applications in the synthesis of functional materials, as well as for controlled drug delivery and bioseparation. Our findings suggest that the stability and responsiveness of fatty acid-based vesicles can be designed by appropriate selection of the chain length of the lipid, the ionic strength of the solution, and the presence of (a wide range of) additives. The reversible and programmable phase transition between lipid bilayers and droplets could be exploited to recruit (bio)molecular cargoes, by exploiting hydrophobic interactions for nonpolar substrates and by functionalizing the lipid–water interface for polar substrates. Moreover, such a versatile method to enable fatty acid phase transitions could potentially be implemented for partitioning macromolecules for purification/separation purposes. Overall, the development of novel methods that trigger downstream reactions by locally increasing the concentration of the reactants would be of interest not only to soft matter scientists and colloid chemists but also for the bottom-up synthetic biology and nanotechnology communities.

Before the evolution of modern lipids and transport systems, primitive cells may have depended on simple physical processes for their replication. In our novel model, we innovatively propose to take advantage of the instability of fatty acids in order to drive recurrent cycles of protocell disassembly and reassembly, in which both the parental membrane material and encapsulated content are transmitted to a new cohort of daughter protocells. Importantly, the prebiotic reshuffling process described herein is likely universal, as it can apply to any protocellular content, including oligonucleotides, long peptides, and membrane-impermeable metabolites. Only the iterative temperature-driven reshuffling of lipid and encapsulated material could have then led to a cascade of new selective pressures for the evolution of more advanced protocells to overcome the intrinsic instability of prebiotic lipids.^39^

If such a primitive membrane-based process could be coupled with nonenzymatic nucleic acid replication, it would set the stage for functional nucleic acids to competitively emerge and support early stages of Darwinian evolution. Additionally, our discovery might hint at a prebiotic means of recycling materials of metabolically “dead” protocells. Hence, the identification of prebiotically relevant disassembly/reassembly cycles like the one proposed herein, supported by mild environmental fluctuations, may represent a step toward the emergence of advanced protocells, which can help in designing artificial systems with life-like behaviors and potentially gaining greater insight into the origin of modern cells.

## Supporting Information Available

Supplementary Movie 1: An epi-fluorescence video showing myristoleic acid (50 mM) vesicles undergoing vesicle-to-droplet conversion upon temperature increase. Similarly, the effect of coalescence at high temperatures and the oil-to-vesicle transition upon temperature decrease can be observed. Scale bar = 10 μm (MP4)
Supplementary Movie 2: An epi-fluorescence video showing myristoleic acid (10 mM) vesicles undergoing vesicle-to-droplet conversion upon temperature increase Similarly, the effect of coalescence at high temperatures and the oil-to-vesicle transition upon temperature decrease can be observed. Scale bar = 10 μm (MP4)
All experimental materials, methods, supplementary Figures S1–S24, and additional references (PDF)

## Notes

A data set in support of this work can be accessed free of charge at 10.17863/CAM.75570.
